# Salinity Tolerance of *Picochlorum atomus* and the Use of Salinity for Contamination Control by the Freshwater Cyanobacterium *Pseudanabaena limnetica*

**DOI:** 10.1371/journal.pone.0063569

**Published:** 2013-05-07

**Authors:** Nicolas von Alvensleben, Katherine Stookey, Marie Magnusson, Kirsten Heimann

**Affiliations:** 1 School of Marine and Tropical Biology, James Cook University, Townsville, Queensland, Australia; 2 Centre for Sustainable Fisheries and Aquaculture, James Cook University, Townsville, Queensland, Australia; 3 Comparative Genomics Centre, James Cook University, Townsville, Queensland, Australia; 4 Centre for Biodiscovery and Molecular Development of Therapeutics, James Cook University, Townsville, Queensland, Australia; Royal Netherlands Institute of Sea Research (NIOZ), The Netherlands

## Abstract

Microalgae are ideal candidates for waste-gas and –water remediation. However, salinity often varies between different sites. A cosmopolitan microalga with large salinity tolerance and consistent biochemical profiles would be ideal for standardised cultivation across various remediation sites. The aims of this study were to determine the effects of salinity on *Picochlorum atomus* growth, biomass productivity, nutrient uptake and biochemical profiles. To determine if target end-products could be manipulated, the effects of 4-day nutrient limitation were also determined. Culture salinity had no effect on growth, biomass productivity, phosphate, nitrate and total nitrogen uptake at 2, 8, 18, 28 and 36 ppt. 11 ppt, however, initiated a significantly higher total nitrogen uptake. While salinity had only minor effects on biochemical composition, nutrient depletion was a major driver for changes in biomass quality, leading to significant increases in total lipid, fatty acid and carbohydrate quantities. Fatty acid composition was also significantly affected by nutrient depletion, with an increased proportion of saturated and mono-unsaturated fatty acids. Having established that *P. atomus* is a euryhaline microalga, the effects of culture salinity on the development of the freshwater cyanobacterial contaminant *Pseudanabaena limnetica* were determined. Salinity at 28 and 36 ppt significantly inhibited establishment of *P. limnetica* in *P. atomus* cultures. In conclusion, *P. atomus* can be deployed for bioremediation at sites with highly variable salinities without effects on end-product potential. Nutrient status critically affected biochemical profiles – an important consideration for end-product development by microalgal industries. 28 and 36 ppt slow the establishment of the freshwater cyanobacterium *P. limnetica*, allowing for harvest of low contaminant containing biomass.

## Introduction

The depletion of fossil energy stores, climate change-associated increasing atmospheric levels of carbon dioxide and freshwater pollution have generated a renewed interest in industrial-scale microalgal biomass production [1]. Industrial algal biomass production can utilize and sequester significant amounts of atmospheric or flue gas carbon dioxide [2] and remove pollutant nutrients such as nitrates, nitrites and phosphates from waste water ponds [3].

To make industrial-scale microalgal cultivation successful, algal strain selection should focus on species with high production of target biochemicals and tolerance to a wide range of environmental conditions, such as salinity, temperature and nutrient or pollutant loads. Such algal ‘super-species’ should also show high biochemical productivity, which would considerably simplify production regarding standardisation of product quality across a range of production sites.

Industry aims for microalgae cultivation at various power-stations in Australia for CO_2_ and NO_x_ remediation from flue gas with parallel production of value-adding biochemicals. However, these sites differ in the water quality for cultivation. A cosmopolitan marine microalga, *Nannochloris atomus* Butcher (Chlorophyta, synonym for *Picochlorum atomus* (Butcher) Henley [4]), has a suitable lipid and protein content for aquaculture [5], [6], high biomass production and a potentially broad tolerance to variations of salinity [7], [8]. However, the influence of salinity on growth patterns, nutrient requirements and biochemical profiles below 36 ppt, which are commonly encountered at potential production sites, have to date not been determined. Establishing species-specific growth parameters will identify optimal inoculation cell numbers and culture durations for achieving highest biomass productivity in the shortest possible timeframe**.** Understanding species-specific daily nutritional requirements will ensure minimal environmental impact (e.g. eutrophication through discharge of nutrient-rich harvest water effluent [9]), whilst also minimising expenses associated with fertilisation.

Nitrate assimilation involves a two-step reduction reaction from nitrate to nitrite and nitrite to ammonium, ultimately resulting in the production of amino acids [10]. Nitrite reduction is rate-limiting and excessive nitrate provision results in an accumulation of cellular nitrite which is secreted, most likely due to its cytotoxicity at high concentrations [11]. This has implications for the remediation of nitric oxide (NO) flue gas, which can be converted 1∶1 to nitrite in water [12] to be then used as a nitrogen source. Similarly, to reduce fertilisation costs, industry aims to remediate nutrient-polluted (waste) waters. Optimal remediation requires correlation of inoculation cell numbers with nutrient loads.

Nitrogen and phosphorus availability also influences cellular protein, carbohydrate, and lipid content, as well as fatty acid profiles [13], [14]. Nitrogen limitation reduces the synthesis of chloroplastic proteins and chl *a*, but increases carotenoid content [15] while the surplus of carbon metabolites are stored as storage lipids and - carbohydrates [13], [16]. Higher lipid yields through nitrogen limitation have been obtained for several microalgal species [13], [17], [18] suggesting that target bio-product yields can be optimised through manipulation of culture nutrient status.

Microalgal culture contamination by rogue organisms is an ever-present risk in aquaculture industries [19]. Common contaminants include bacteria, viruses, fungi, other algae and zooplankton (e.g. ciliates, copepods, rotifers) [20]. Current procedures to minimise culture contamination include pH or salinity manipulations [19], [20], the use of ammonium as a nitrogen source, or quinine treatment to reduce amoeba populations [20], [21]. Other remedies, such as the addition of antibiotics [22] carry the risk of antibiotic resistance, placing restrictions on the use of the biomass and waste water disposal.

Culture contamination by non-target algae or cyanobacteria generally results in resource competition [23] and/or the release of potentially toxic allelochemicals into the culture medium, inhibiting growth or killing the target species [24]. This often leads to lost productivity associated with disposal of contaminated cultures, sterilisation, re-inoculation and culture re-establishment. Adverse impacts on product quality can further negatively affect industry, even if productivity is unaffected.

*Pseudanabaena limnetica* (Lemmermann) Komàrek is a filamentous, non-heterocystous [25] and non-toxic [26] freshwater cyanobacterium [27], with a certain degree of halotolerance [28] and is a frequent local nuisance contaminant in outdoor microalgal cultures during the tropical wet season. Consequently, methods must be developed to control levels of contamination, ideally not affecting the target species or influencing final products.

Given the potential importance of *P. atomus* in aquaculture, this study firstly aimed to determine the influence of salinity on growth, nutrient utilisation, biomass and lipid production and effects of nutrient limitation on biochemical profiles to determine end-product choice and industrial-scale cultivation protocols. Additionally, the effectiveness of salinity manipulations for contamination control of the freshwater cyanobacterial contaminant *P. limnetica* was investigated.

It is shown that salinity had no effect on *P. atomus* growth and nutrient utilisation (except at 11 ppt for the latter) and had only a marginal effect on total lipid at 2 ppt and carbohydrate at 8 ppt, respectively, under nutrient-replete conditions. Nutrient status, however, significantly affected total lipid and fatty acid profiles, carbohydrate and protein contents. It is further shown that salinity can be used to control the establishment of *P. limnetica*.

## Materials and Methods

### Algal culture conditions

Batch cultures of *Picochlorum atomus* (culture accession # NQAIF 284) were maintained (24 °C, with a 12∶12 h photoperiod and light intensity of 42 µmol photons m^−2^ s^−1^) at the North Queensland Algal Identification/Culturing Facility (NQAIF) culture collection (James Cook University, Townsville, Australia), and were individually areated with 0.45 µm filtered air (Durapore; Millipore). Monoclonal cultures with low bacterial numbers (<1 mL^−1^) were established in a total culture volume of 2 L in modified L1 culture medium [29], with 6 mg instead of 3 mg PO_4_^3−^ L^−1^. Cultures were re-fertilised with nitrate (∼55 mg L^−1^) and phosphate (6 mg L^−1^) on day 5 after inoculation to generate sufficient biomass for biochemical analyses of nutrient-replete cultures.

The modified L1 culture medium was prepared at six different salinities: 2, 8, 11, 18, 28 and 36 ppt NaCl in filtered seawater (FSW) (pre-filtration Whatman GF/C, followed by 0.45 µm Durapore, Millipore). All materials were sterilised by autoclaving (Tomy, Quantum Scientific) and cultures were handled and inoculated aseptically in a laminar flow (AES Environmental Pty LTD fitted with HEPA filter). Replicate cultures (2 L, n = 3) of *P. atomus* were inoculated at a density of 4×10^9^ cells L^−1^ (∼100 mg dry weight L^−1^) for each salinity. Inoculation was carried out from 36 ppt mother-cultures with no acclimation to decreasing salinity. Cultures of *P. atomus* have been maintained at the above salinities for more than 200 generations showing the same growth and nutrient utilisation patterns.

### Indirect methods for culture growth determination

Calibration curves were established from triplicate dilution series using *Picochlorum atomus* stock cultures to correlate cells L^−1^ (direct cell counts using a bright-line Neubauer-improved haemocytometer) and dry weights (DW) [g L^−1^] (gravimetric analysis, modified from Rai et al. [30]) with turbidity (% transmission [% TA at 750 nm, Spectramax Plus; Molecular Devices]). Turbidity and calibration curves were medium blanked for each salinity. Dry weight samples were corrected for salt content using salinity-specific blanks. Results were correlated to generate linear equations (R^2^ >0.95) used to determine cell numbers and respective dry weights of cultures of *P. atomus* from turbidity measurements.

### Culture growth and nutrient analysis

Growth of *Picochlorum atomus* was determined daily using turbidity, from triplicate 250 µl samples per culture for 20 days and obtained data were transformed to cell numbers and dry weights as described above. Specific growth rates [µ], (eq. 1) were calculated from culture cell numbers [31], as were the derived parameters divisions per day and generation time [days]. Biomass productivies were determined using equation 2 (modified from Su et al. [32]).



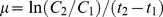
(eq. 1)




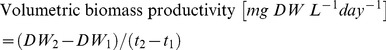
(eq. 2)


where C_1_ and C_2_ =  initial and final cell numbers [cells mL^−1^], respectively, t_1_ and t_2_ =  initial and final culture timepoints [days] per identified growth period, respectively, DW_1_ and DW_2_  =  initial and final dry weight [g L^−1^].

Medium nitrate (NO_3_^−^), nitrite (NO_2_^−^) and phosphate (PO_4_^3−^) concentrations were determined every second day and on day 5, following nutrient addition, using the Systea EasyChem (Analytical Solutions Australia (ASA)) auto-analyser following the manufacturer’s EPA-approved and certified protocols (Systea User Manual, 2011).

### Biochemical analyses

#### Total lipids, FAME, carbohydrate and protein

Biomass samples for biochemical analyses were harvested from 500 mL samples through centrifugation (20 min at 3000 g (Eppendorf 5810R), followed by 2 min at 16,000 g (Sigma 1–14, John Morris Scientific)) from all cultures when nitrate-replete during late logarithmic growth (day 11) and four days after nitrogen depletion during the initial stationary phase; i.e. days 18 and 22, for cultures at 11 and 2 ppt, respectively, and day 24 for cultures at 8, 18, 28 and 36 ppt. Cultures were classified as nutrient-replete and -deplete based on increasing and decreasing nitrite secretion patterns and the nutrient depletion was assured by harvesting four days after medium nutrient depletion [33]. The biomass pellets were lyophilized (VirTis benchtop 2K, VWR) and stored in air-tight vials under nitrogen at 4 °C until further analysis.

### Total lipid determination

Total lipids were determined gravimetrically following a direct extraction and transesterification method adapted from Lewis et al. [34] and modified following Rodriguez-Ruiz et al. [35], and Cohen et al. [36]. Briefly, 2 mL freshly prepared methylation reagent (HPLC-grade methanol : acetyl chloride, 95∶5 (v/v)) and 1 mL hexane were added to 30±0.1 mg lyophilized biomass. Following heating (100°C, 60 min), 1 mL MilliQ purified water was added and the samples were centrifuged (1800 g for 5 min at 4°C (Eppendorf 5810R, VWR) to achieve phase separation. The hexane layer was collected and the pellet was extracted twice more with 1 mL hexane, centrifuging as above between washes, to extract all lipids into the organic phase. The hexane extracts were combined (total of 3 mL) in a pre-weighed glass vial and evaporated to dryness under a gentle stream of nitrogen and weighed to determine total lipids.

### Fatty acid extraction, transesterification and analysis

Fatty acids in lyophilised samples were simultaneously extracted and transesterified using a method adapted from Rodriguez-Ruiz et al. [35] and Cohen et al. [36] as described in Gosch et al. [37]. Fatty acid analysis was carried out on an Agilent 7890 GC (DB-23 capillary column with a 0.15 µm cyanopropyl stationary phase, 60 m×0.25 mm ID (inner diameter)) equipped with flame ionisation detector (FID) and connected to an Agilent 5975C electron ionisation (EI) turbo mass spectrometer (Agilent technologies), for identification of fatty acid methyl esters (FAMEs) (split injection, 1/50). Injector, FID inlet and column temperatures were programmed following David et al. [38]. Fatty acid quantities were determined by comparison of peak areas of authentic standards (Sigma Aldrich) and were corrected for recovery of internal standard (C19∶0) and total fatty acid content (mg g^−1^ DW) was determined as the sum of all FAMEs.

### Total lipid and FAME productivities

Total fatty acid productivities were determined using equation 3, where total FAME_2_ was determined in nutrient-deplete conditions, total FAME_1_ in nutrient-replete conditions, and t_1_ and t_2_  =  harvest time points for FAME determination.



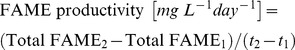
(eq. 3)


### Carbohydrate analysis

Total carbohydrate content was determined using the phenol-sulphuric acid assay [39]. Prior to analysis, lyophilised algal samples were lysed in MilliQ-purified water with a Bullet Blender bead beater (ZrO_2_ beads, 0.5 mm diameter) (Next Advance, Lomb Scientific) to enable collection of a homogenous sub-sample for extraction.

### Ash and protein analysis

Ash-content (mg g^−1^ DW) was determined by combustion in air (500°C, 24 h) (Yokogawa-UP 150, AS1044) while protein content was determined by difference (eq. 4) [40].



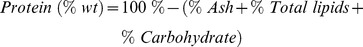
(eq. 4)


### Effect of salinity on contamination of *Picochlorum atomus* cultures with *Pseudanabaena limnetica*

To investigate if salinity could be used for contamination control, cultures of *Picochlorum atomus* were raised at 11, 18, 28 and 36 ppt (cultures at 2 and 8 ppt were not established as *P. limnetica* is a freshwater species) and seeded with *Pseudanabaena limnetica* colonies at a ratio of 1∶100,000 cells mL^−1^ (*P. limnetica* : *P. atomus*). Cell counts (bright-line Neubauer improved haemocytometer) of both organisms commenced on day 8 after the first visible signs of *P. limnetica* dominance (culture colour change) in the lower salinity cultures (11 and 18 ppt).

### Statistical analyses

All statistical analyses were carried out in Statistica 10 (StatSoft Pty Ltd.). Repeated measures ANOVAs were used to determine the effects of salinity on growth rates, nitrite secretion, total nitrogen uptake and contaminant development through culture time. One-way ANOVAs were used to determine the effect of salinity on volumetric biomass productivities. For nutrient uptake analyses, data were divided into pre- and post- nutrient addition (days 0–4 and 5–10, respectively) and the slopes of each uptake period were analysed using one-way ANOVAs. For total lipid, fatty acids, carbohydrate and protein content analyses, factorial ANOVAs were used to determine the effects of salinity, nutrient status and their interaction. Tukey post-hoc tests were used to determine significant differences assigned at p< 0.05. Homogeneity of variances and normality assumptions were verified using Cochran-Bartlett tests. Fatty acid and carbohydrate data required log transformation to fulfil normality assumptions.

## Results

### Effect of salinity on growth and nutrient uptake dynamics of *Picochlorum atomus*

**Figure 1:**
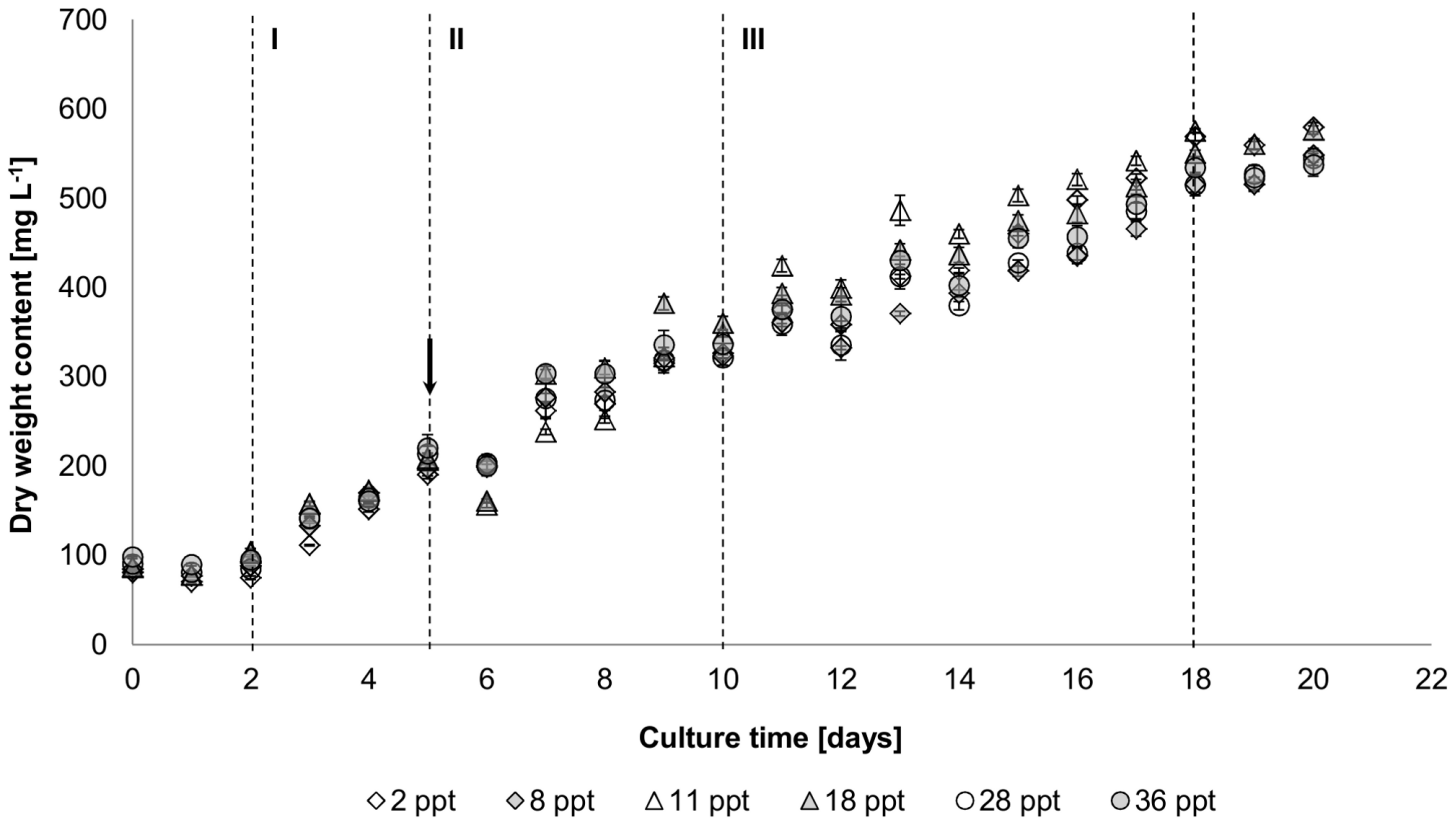
Mean biomass growth [mg DW L^−1^] of *Picochlorum atomus* at 2, 8, 11, 18, 28 and 36 ppt determined using% transmittance at 750 nm. Arrow: indicates the addition of nutrients. Active growth was divided into 3 phases (I–III) based on log-transformed data for determination of specific growth rates [µ]. n = 3. Standard error is shown. DW: dry weight.

Culture growth of *P. atomus* was divided into three phases (phase I: days 2–5; phase II: days 5–9 and phase III: 9–18) (Fig. 1) for which specific growth rates, divisions per day and generation times were calculated (Table 1). Within each growth phase, salinity had no significant effect (F_(5, 12)_ = 0.99, p = 0.46) on growth rates, while the effect of culture phase was significant (F_(2, 24)_ = 679.67, p<0.01) as growth rates decreased over culture time. Irrespective of salinity, specific growth rates [µ] were highest for the first two days following a one-day lag phase (µ = 0.21–0.28), then decreased by ∼50% during phase II and a further ∼50% thereafter during phase III (Table 1). Nutrient addition on day 5 resulted in culture dilution (Fig. 1).

**Table 1:** Effect of salinity on specific growth rates [μ], divisions per day and generation times [days] of *Picochlorum atomus.*

Culture time [days]	2 ppt growth rate [µ]	8 ppt growth rate [µ]	11 ppt growth rate [µ]	18 ppt growth rate [µ]	28 ppt growth rate [µ]	36 ppt growth rate [µ]
Days 2–5	0.28	0.25	0.21	0.27	0.28	0.26
Days 5–9	0.13	0.13	0.11	0.14	0.11	0.11
Days 9–18	0.06	0.05	0.06	0.04	0.05	0.05

Culture time [days]	2ppt [Div. day^−1^]	8 ppt [Div. day^−1^]	11 ppt [Div. day^−1^]	18 ppt [Div. day^−1^]	28 ppt [Div. day^−1^]	36 ppt [Div. day^−1^]
Days 2–5	0.4	0.35	0.3	0.39	0.4	0.37
Days 5–9	0.19	0.18	0.17	0.2	0.15	0.16
Days 9–18	0.09	0.07	0.09	0.06	0.07	0.07

Culture time [days]	2 ppt generation time [days]	8 ppt generation time [days]	11 ppt generation time [days]	18 ppt generation time [days]	28 ppt generation time [days]	36 ppt generation time [days]
Days 2–5	2.47	2.82	3.29	2.58	2.46	2.69
Days 5–9	5.39	5.51	6.04	4.94	6.6	6.35
Days 9–18	11.15	14.08	11.35	18.19	13.85	14.2

Biomass productivities during growth phase I were between 34–43 mg L^−1^ day^−1^ and 26–31 mg L^−1^ day^−1^ during phase II, with the exception of cultures at 18 ppt where biomass productivity remained similar at 36 mg L^−1^ day^−1^ (Table 2). Productivities, from the beginning of the logarithmic growth phase to the beginning of the stationary phase were approximately 27–30 mg L^−1^ day^−1^.

**Table 2:** Effect of salinity on volumetric biomass productivities of *Picochlorum atomus* during growth phases I and II, and overall from days 2–18.

	Total dry-weight productivity [mg DW L^−1^ day^−1^]					
Culture phase	2 ppt	8 ppt	11 ppt	18 ppt	28 ppt	36 ppt
**Days 2**–**5**	38±1	37±3	34±1	35±1	43±2	42±3
**Days 5**–**9**	31 ±1	31±1	29±1	36±1	26 ±1	28±1
**Days 2**–**18**	30±0.5	27±0.5	29±0.5	28±0.5	27±0.5	27±0.5

n = 3. Average ± standard error.

**Figure 2:**
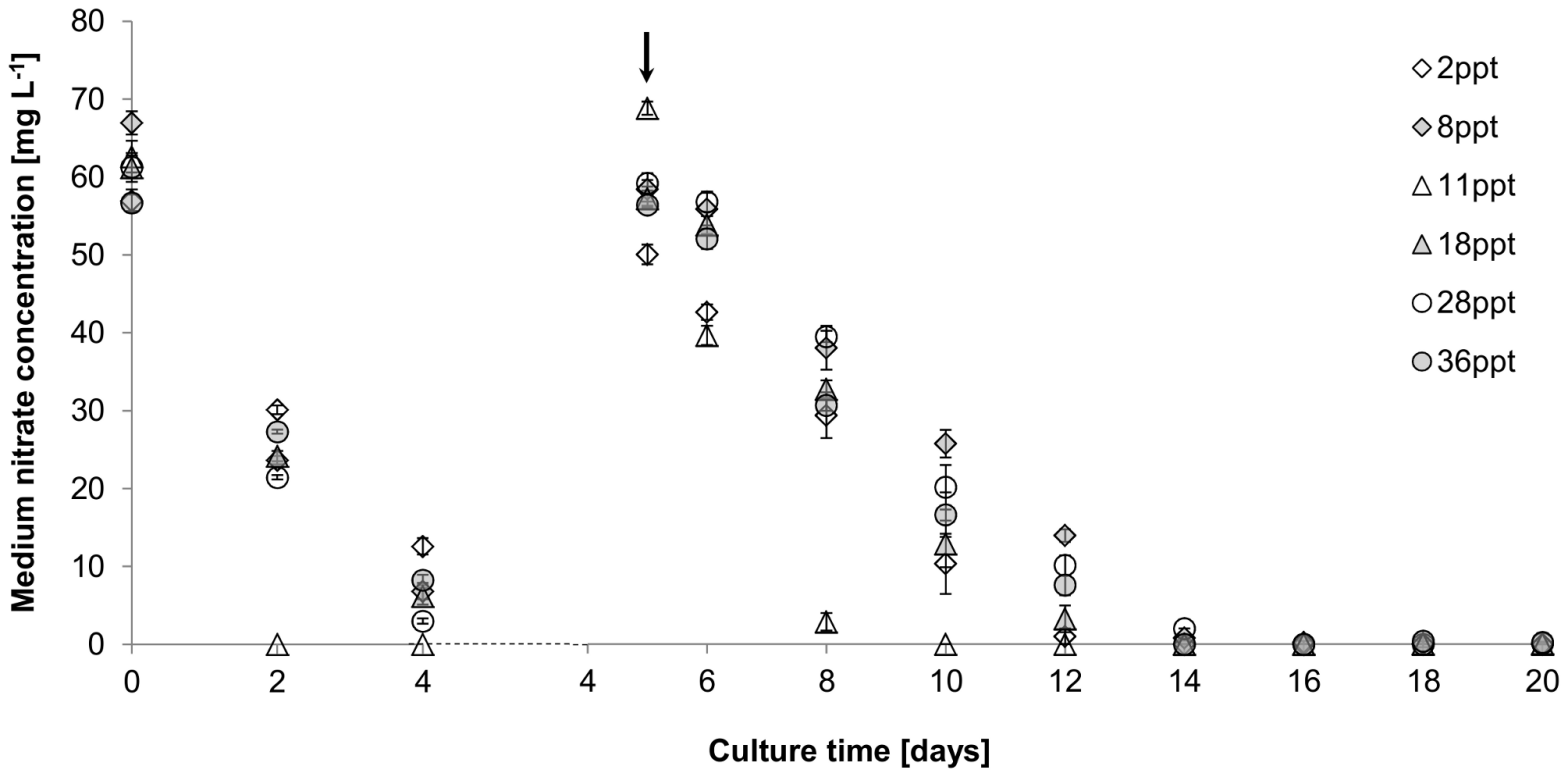
Effect of salinity on nitrate assimilation [mg L^−1^] of *Picochlorum atomus*. n = 3. Standard error is shown. Arrow: indicates measurements taken after nitrate and phosphate replenishment.

Except for cultures at 11 ppt, salinity had no effect on nitrate uptake of *P. atomus* for the first 4 days of the culture period with ∼13–15 mg nitrate L^−1^ day^−1^ being assimilated. Following nutrient replenishment on day 5, a ∼50% decrease in nitrate uptake was observed (Fig. 2). Cultures at 11 ppt took up nitrate significantly faster pre- (F_(5, 12)_ = 85.48, p<0.01) and post- (F_(5, 12)_ = 14.68, p<0.01) fertilisation, than cultures at the other salinities resulting in an uptake of 60 mg L^−1^ day^−1^ for the first two days and medium nitrate depletion.

**Figure 3:**
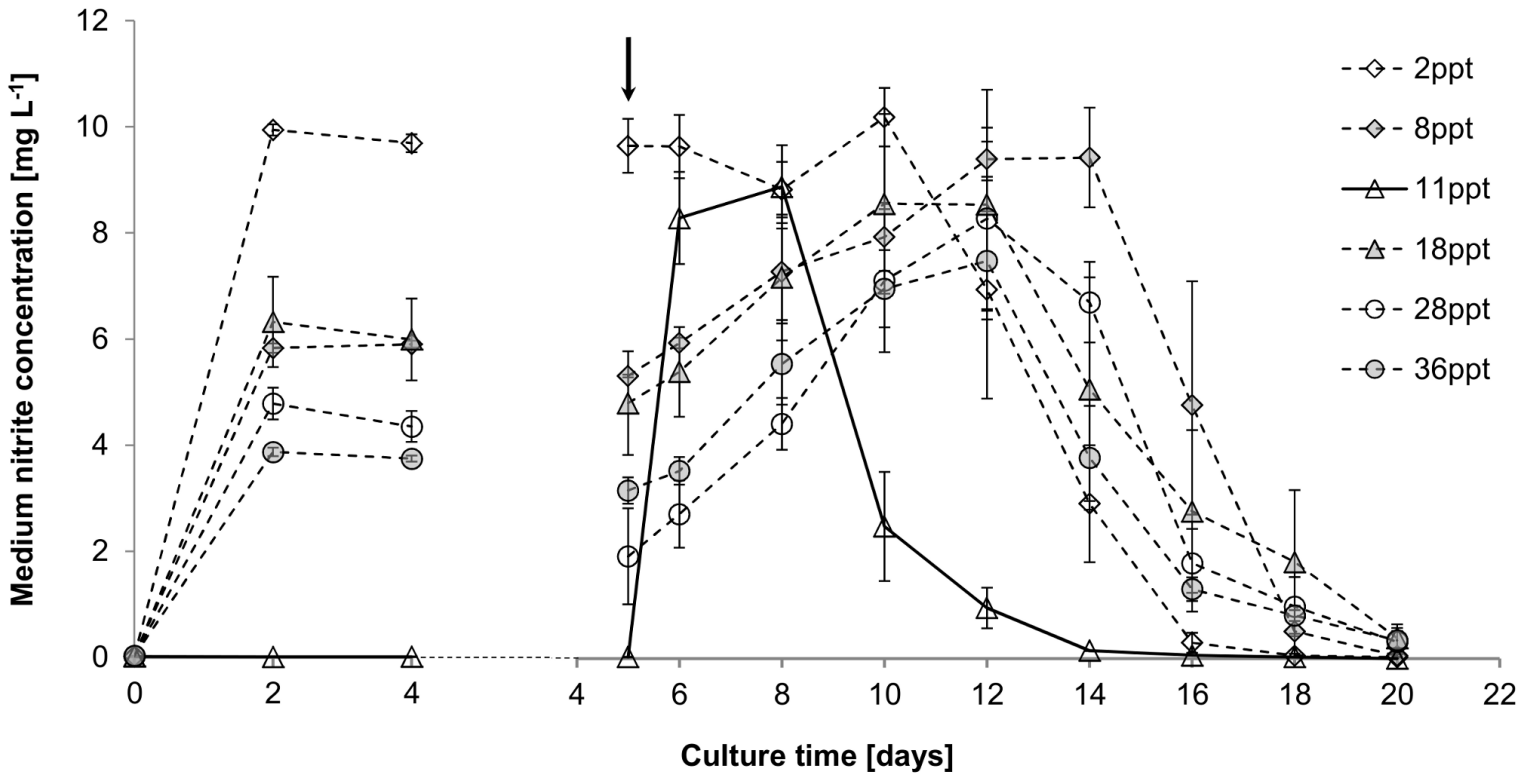
Effect of salinity on media nitrite dynamics [mg L^−1^] of *Picochlorum atomus*. n = 3. Standard error is shown. Arrow: indicates measurements taken after nitrate and phosphate replenishment.

**Figure 4:**
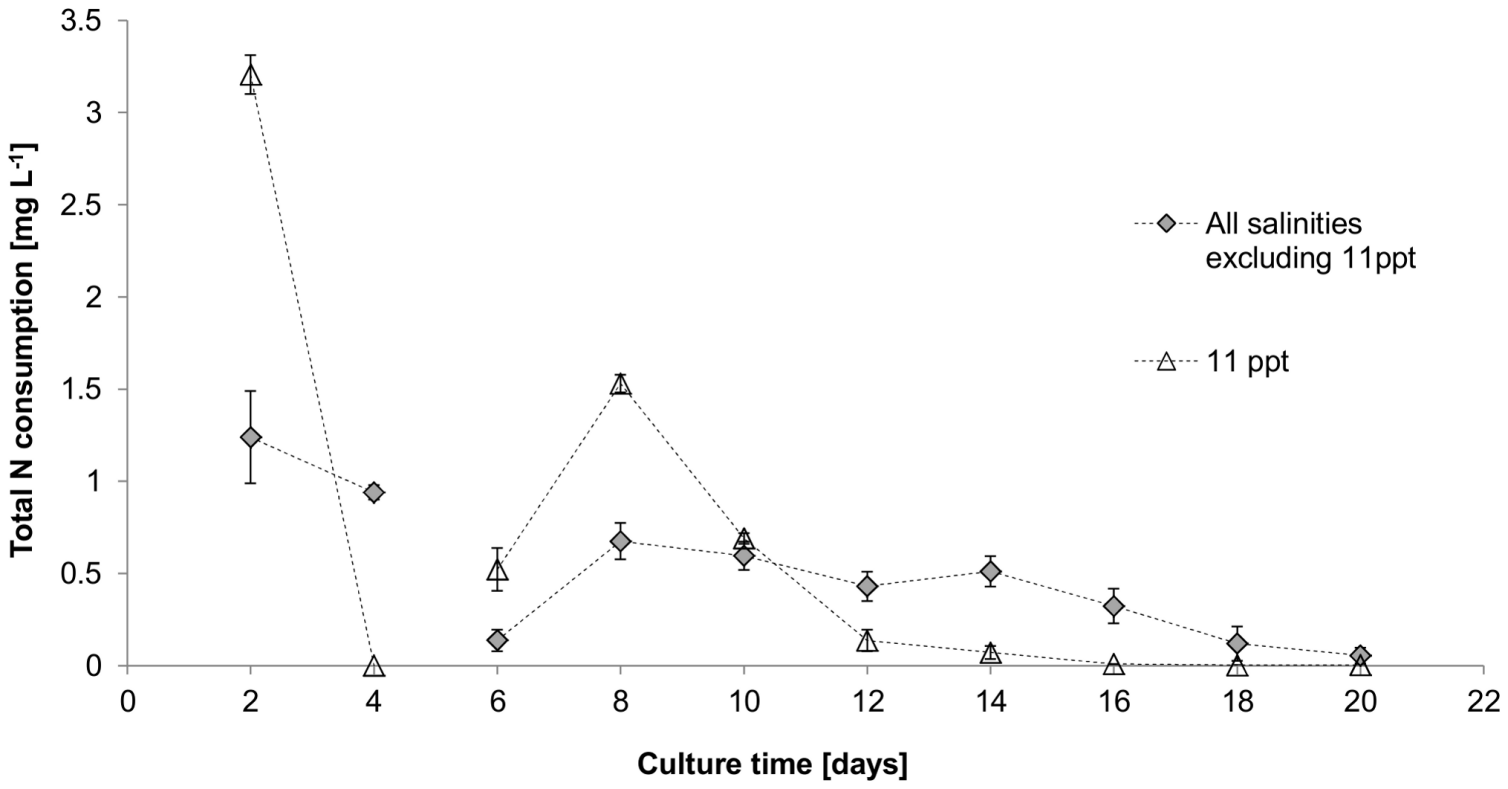
Total daily net nitrogen [N] uptake [mg L^−1^] of *Picochlorum atomus*. Average total nitrogen consumption is shown for salinities of 2, 8, 18, 28 and 36 ppt, while nitrogen consumption of cultures at 11 ppt is plotted individually to highlight the effect of 11 ppt. n = 3. Standard error is shown.

In contrast, a significant negative correlation between nitrite release and salinity (F_(5, 12)_ = 6.13, p<0.05) was observed prior to re-fertilisation, except for cultures at 11 ppt which showed no nitrite release (Fig. 3). Following fertilisation, all cultures released nitrite irrespective of salinity. Nitrite resorption started 4, 6, 10 and 12 days after fertilisation for cultures at 11 ppt, 2 ppt, 18 and 36 ppt, and 8 ppt, respectively, which correlated with medium nitrate depletion in most cultures (compare Fig. 2 and Fig. 3). Total daily nitrogen uptake (Fig. 4) was similar between cultures at 2, 8, 18, 28 and 36 ppt but significantly higher at 11 ppt (F _(5, 12)_  = 34.079, p<0.01).

**Figure 5:**
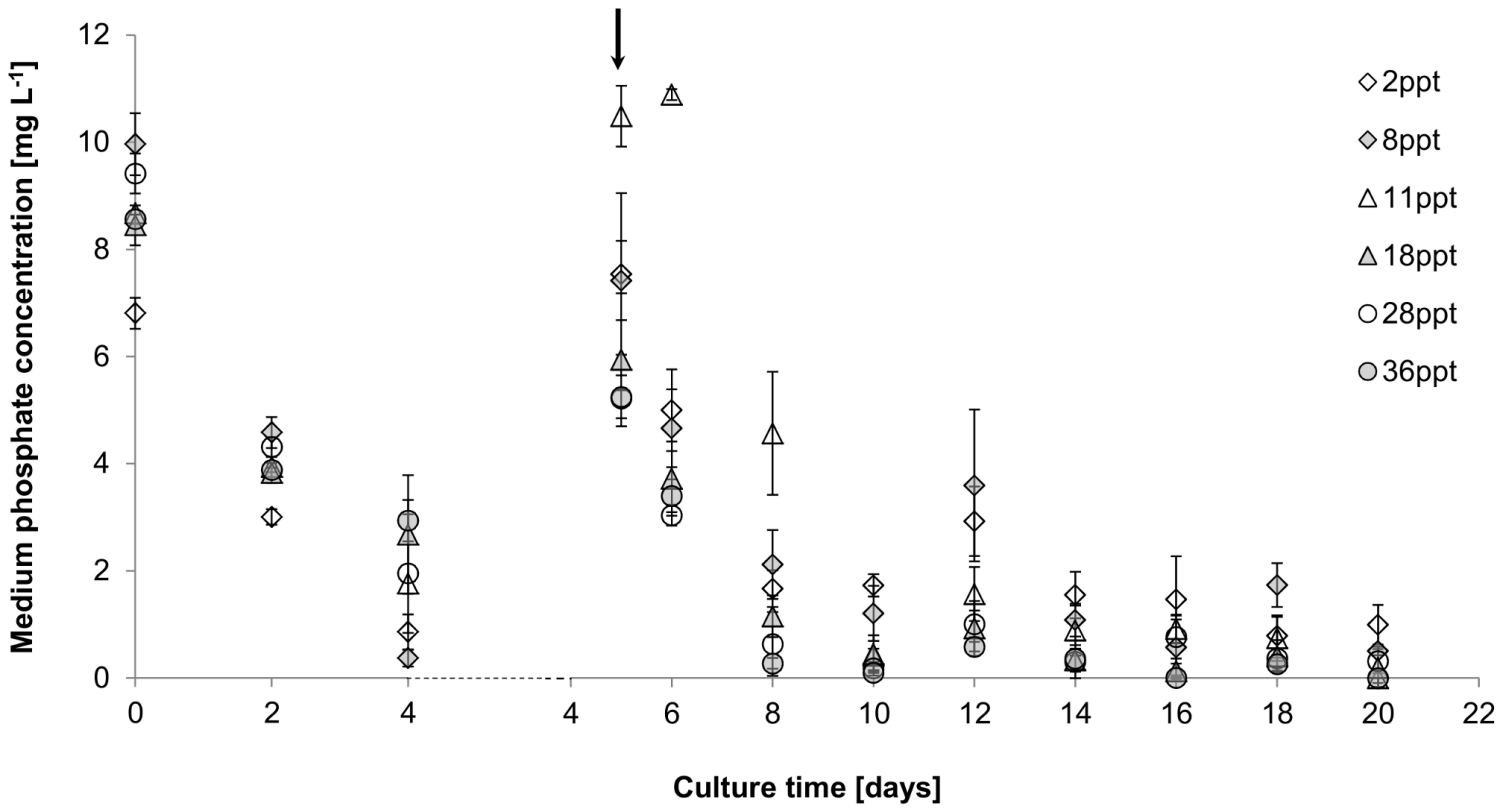
Effect of salinity on phosphate assimilation [mg L^−1^] of *Picochlorum atomus*. n = 3. Standard error is shown. Arrow: indicates measurements taken after nitrate and phosphate replenishment.

Phosphate uptake followed a similar pattern to nitrate with a decrease in uptake rates following fertilisation. Initial phosphate uptake rates were 1.3–2.4 mg L^−1^ day^−1^ (Fig. 5). Following phosphate addition, uptake rates decreased to 0.8–1 mg L^−1^ day^−1^, except for cultures at 11 ppt. Initially, nitrate to phosphate uptake ratio was 6–9:1 (N:P) and decreased to 4–7:1 (N:P) after nutrient addition.

### Effect of salinity and culture nutrient status on the biochemical profile of *Picochlorum atomus*

**Figure 6:**
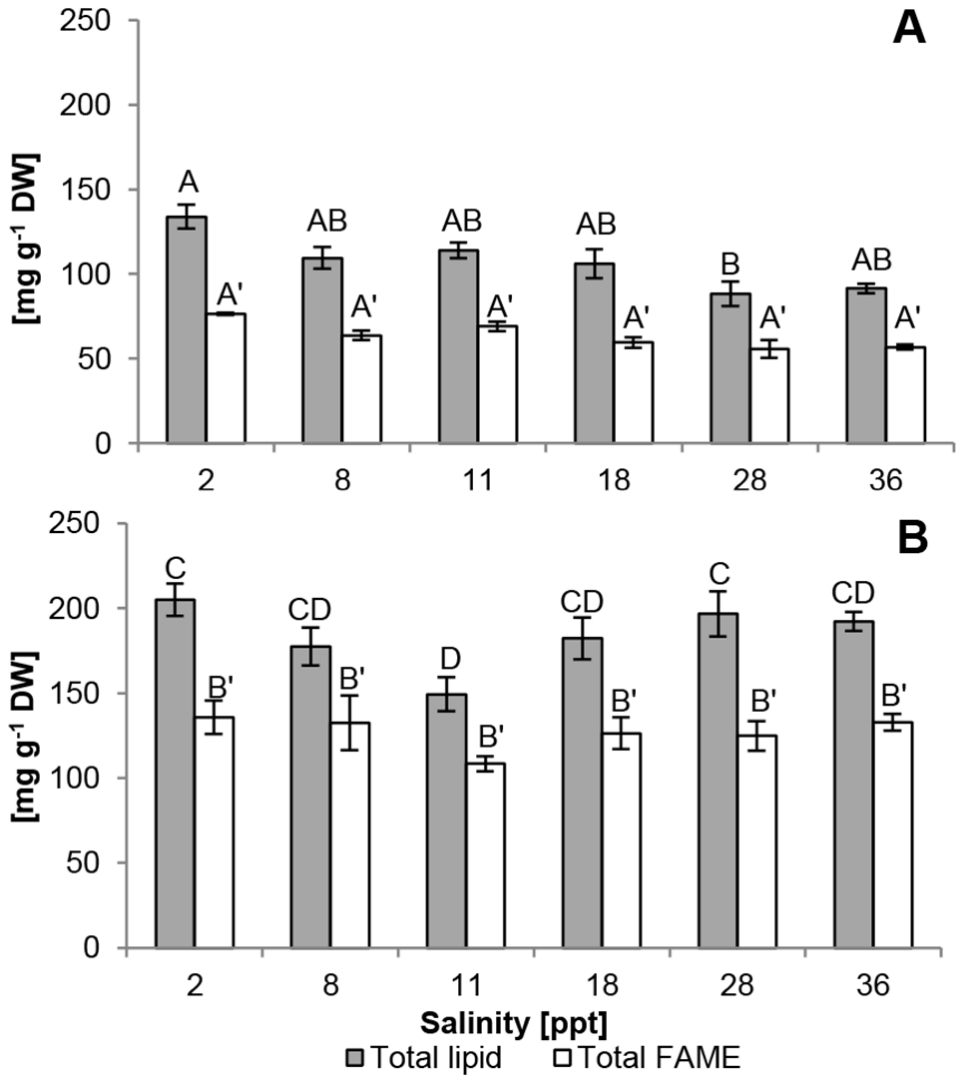
Effect of nutrient availability and salinity on total lipid and fatty acid content. Total lipid and FAME content under nutrient replete conditions (A) and nutrient deplete conditions (B). Grey bars: total lipid, white bars: total FAME. n = 3. Standard error is shown. Statistically significant differences are indicated by different letters. Prime letters are used for total FAME.

Post hoc analyses identified marginally significant effects of salinity on total lipid content of *P. atomus* at 2 ppt compared to 28 and 36 ppt under nutrient-replete conditions (Fig. 6A), whereas culture nutrient status had a large effect (F_ (1, 24)_  = 229.63, p<0.01). Nutrient-starved cultures of *P. atomus* had significantly higher lipid content (F_ (1, 24)_  = 229.63, p<0.01) than nutrient-replete cultures (Fig. 6). After 4 days of nutrient starvation, biomass total lipid content increased by 3.5–11% with the lowest increase in cultures at 11 ppt and the highest increase in cultures at 28 and 36 ppt (Fig. 6).

There was no significant effect of salinity on total fatty acid content, but there was a significant effect of culture nutrient status (F_ (1, 24)_  = 4.42, p<0.01) where, as with lipid content, total fatty acid content in nutrient-deplete cultures was significantly higher than in replete biomass.

Fatty acids represented 56–66% of total lipids in nutrient-replete biomass and 66–74% of total lipids in nutrient-deplete cultures, with cultures at 2 ppt showing the highest fatty acid content under both nutrient conditions (Fig. 6). Lowest fatty acid concentrations were recorded in nutrient-replete cultures at 28 ppt and 36 ppt (Fig. 6A). Fatty acid productivities between nutrient-replete and -deplete conditions ranged from 4.7–6.2 mg L^−1^ day^−1^ with cultures at 11 ppt and 2 ppt showing the lowest and highest productivities, respectively (Table 3).

**Table 3:** Total FAME productivities [mg L^−1^ day^−1^] of *Picochlorum atomus* from nutrient replete to deplete conditions.

Salinity	Total FAME productivity [mg L^−1^ day^−1^]
2 ppt	6.2±0.25
8 ppt	6.1±0.13
11 ppt	4.7±0.06
18 ppt	6.0±0.09
28 ppt	5.9±0.16
36 ppt	6.2±0.13

n = 3. Average ± standard error.

While fatty acid content increased by up to 50% following 4 days of nutrient starvation (Fig. 6), nutrient status also had an influence on fatty acid profiles. A 9 and 11% increase in saturated and mono-unsaturated fatty acids, respectively, and a corresponding decrease in polyunsaturated fatty acids was observed in nutrient-starved *P. atomus* cultures (Table 4). Specifically, C18∶1 increased by ∼ 13% while C18∶3 showed the largest decrease. The most abundant fatty acids were always C18∶3 (n-3), C16∶0, and C18∶2 (n-6), equating to 54–68% of the total fatty acids (Table 4). The observed ∼50% decrease in the proportion of omega-3 fatty acids and a small increase of omega-6 fatty acids led to a change in omega-6 to omega-3 ratios (ω6∶ω3) from ∼0.5∶1 to ∼1∶1 under nutrient-limiting conditions.

**Table 4:** Effect of salinity and culture nutrient status (replete/deplete) on fatty acid profiles (proportion [%] of total FAME) of *Picochlorum atomus*.

	2 ppt		8 ppt		11 ppt		18 ppt		28 ppt		36 ppt	
	Replete	Deplete	Replete	Deplete	Replete	Deplete	Replete	Deplete	Replete	Deplete	Replete	Deplete
**Saturated**												
C12∶0	0.24	0.16	0.48	0.20	0.84	0.45	0.38	0.19	0.50	0.24	0.40	0.21
C14∶0	0.38	0.47	0.45	0.53	0.50	0.51	0.46	0.59	0.48	0.56	0.45	0.63
C16∶0	14.95	22.50	15.00	22.22	15.68	20.86	15.38	21.69	15.27	21.81	15.82	21.54
C18∶0	1.08	4.56	1.20	5.00	1.29	2.75	1.11	3.84	1.32	4.48	1.24	3.74
C20∶0	2.21	1.85	2.22	1.94	2.44	1.95	2.29	1.96	2.86	1.94	2.88	2.00
Σ*_SFA_*	18.86	29.55	19.35	29.89	20.76	26.51	19.62	28.27	20.42	29.03	20.79	28.11
**Monounsaturated**												
C16∶1 (7)	1.03	1.26	0.93	1.05	1.44	1.26	0.99	1.07	1.09	0.93	0.98	0.85
C16∶1 (9)	3.25	1.20	3.17	1.11	3.39	1.59	3.03	1.22	2.89	1.10	2.97	1.24
C18∶1 (9)	1.82	14.76	1.99	17.01	4.50	11.63	1.68	14.21	2.07	19.13	1.95	17.96
C18∶1 (x)	0.87	0.79	1.03	0.87	1.28	1.12	1.01	0.88	1.17	1.26	1.16	1.43
Σ*_MUFA_*	6.99	18.01	7.12	20.04	10.61	15.60	6.71	17.39	7.22	22.43	7.06	21.48
**Polyunsaturated**												
C16∶2 (7,10)	8.80	6.47	7.56	5.64	7.29	6.42	8.15	6.45	7.36	5.16	6.89	5.08
C16∶2 (9, 12)	0.37	0.40	0.39	0.39	0.37	0.33	0.37	0.42	0.45	0.40	0.48	0.34
C16∶3 (7,10,13)	12.35	6.15	12.81	6.18	9.76	7.47	12.08	6.57	11.69	5.22	11.70	5.82
C16∶4 (4,7,10,13)	0.77	0.35	0.83	0.35	0.67	0.41	0.79	0.35	0.79	0.30	0.81	0.30
C18∶2	14.26	18.93	12.36	17.20	15.39	17.84	13.23	19.15	13.30	19.31	13.18	19.31
C18∶3 (6,9,12)	0.44	0.22	0.40	0.23	0.43	0.30	0.51	0.25	0.48	0.23	0.49	0.23
C18∶3 (9,12,15)	34.62	18.48	36.09	18.59	31.90	23.29	35.27	19.60	34.80	16.29	35.19	17.83
*Σ_PUFA_*	71.60	50.99	70.44	48.58	65.80	56.06	70.40	52.78	68.87	46.91	68.75	48.90
Sum of ω3	47.74	24.98	49.73	25.12	42.32	31.17	48.14	26.51	47.28	21.81	47.70	23.94
Sum of ω6	23.49	25.62	20.31	23.07	23.11	24.56	21.89	25.85	21.14	24.70	20.56	24.62
ω6∶ω3 ratio	0.49	1.03	0.41	0.92	0.55	0.79	0.45	0.97	0.45	1.13	0.43	1.03

n = 3.

**Figure 7:**
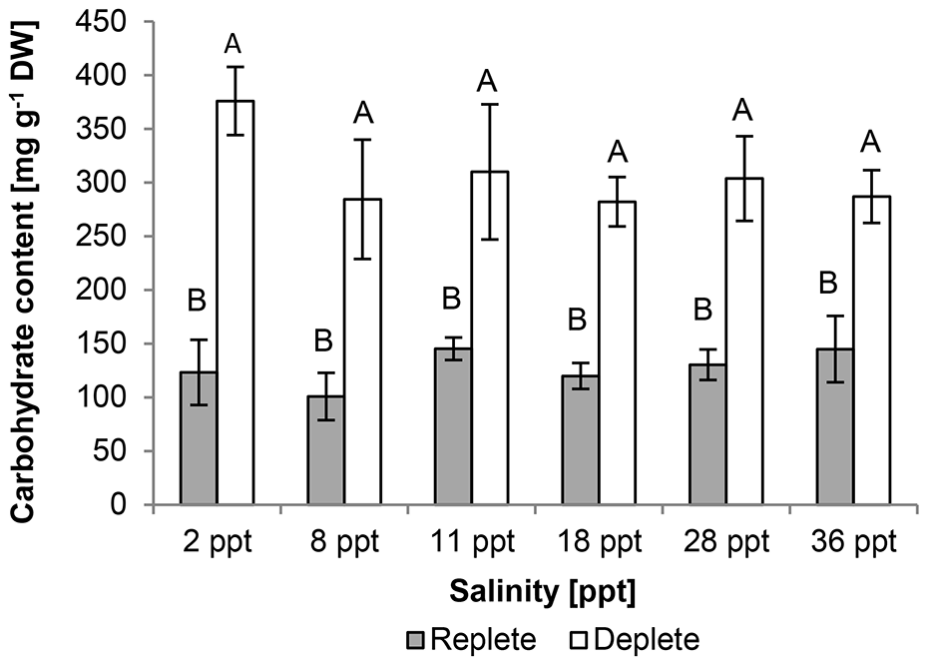
Effect of salinity and culture nutrient status (replete/deplete) on mean carbohydrate content [mg glucose g^−1^ DW] of *Picochlorum atomus*. n = 3. Standard error is shown. Different letters show statistical significance.

Carbohydrate contents were 120–250 mg g^−1^ DW in nutrient-replete cultures, with cultures at 2 ppt and 36 ppt containing the lowest and highest concentrations, respectively. Overall, cellular carbohydrate contents were not affected by salinity, but did increase two to three-fold across all salinities in nutrient-deplete cultures (F_ (1, 24)_  = 86.98, p<0.01) (Fig. 7).

**Figure 8:**
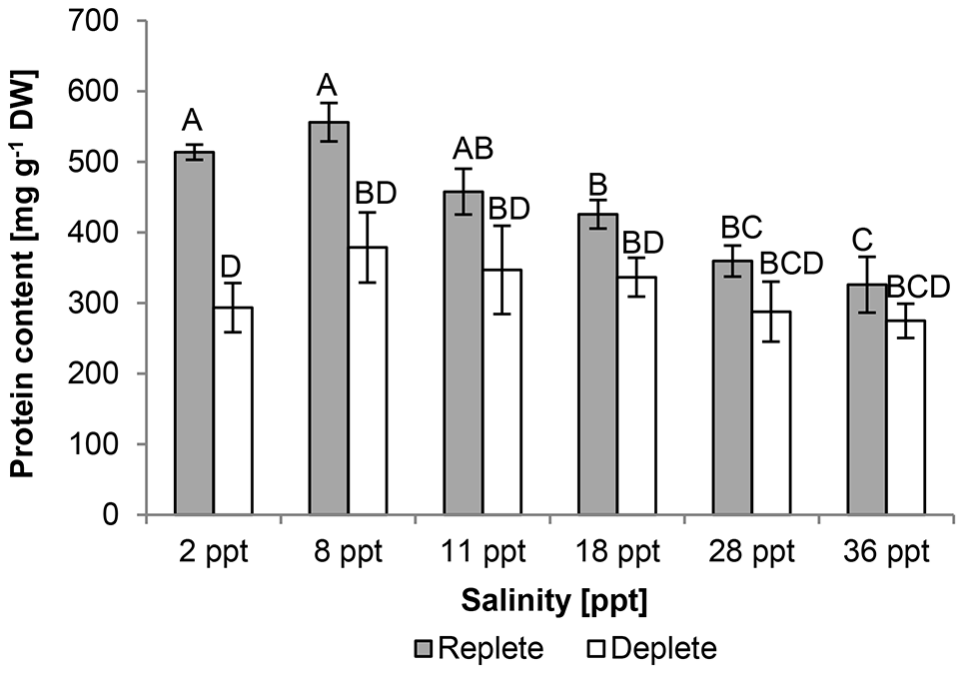
Effect of salinity and culture nutrient status (replete/deplete) on mean protein content [mg protein g^−1^ DW] of *Picochlorum atomus*. n = 3. Standard error is shown. Different letters show statistical significance.

Ash content increased with increasing salinity irrespective of nutrient status. Nutrient depletion led to a ∼50% decrease in ash content compared to replete cultures. Protein content was significantly higher (F_ (5, 24)_  = 5.78, p < 0.01) in cultures at 8 ppt compared to cultures at 28 and 36 ppt in nutrient-replete conditions. Nutrient depletion induced a protein content decrease across all salinities with a significant decrease in cultures at 2 ppt (∼40%) and 8 ppt (∼30%) (F_ (1, 24)_  = 34.34, p<0.01) (Fig. 8). In both nutrient-replete and -deplete conditions, 8 ppt cultures had the highest protein content and cultures at 36 ppt the lowest.

### Effect of salinity on contamination of *Picochlorum atomus* cultures with *Pseudanabaena limnetica*

**Figure 9:**
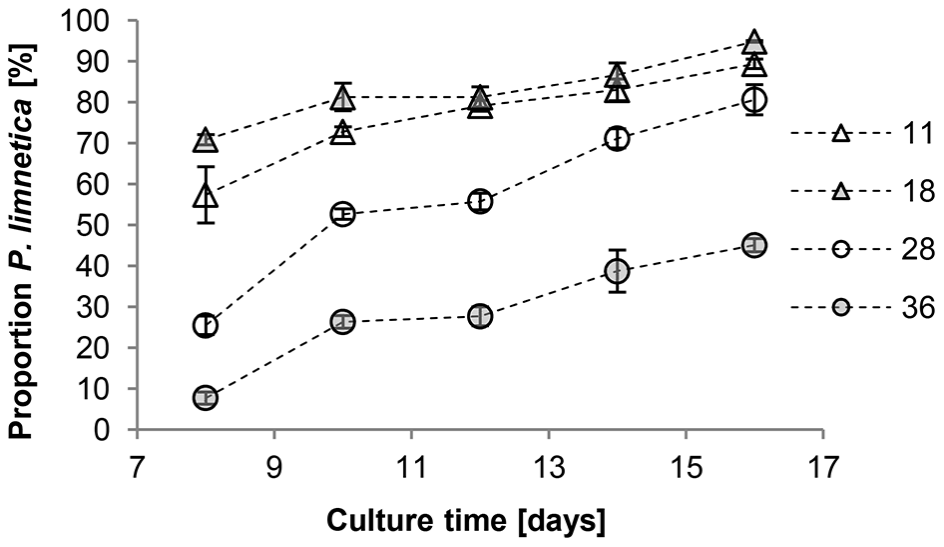
Effect of salinity (11, 18, 28 and 36 ppt) on the proportion [%] of *Pseudanabaena limnetica* in *Picochlorum atomus* cultures. n = 3. Standard error is shown.

An increase in salinity significantly (F_ (15, 40)_  = 5.7, p<0.01) slowed the establishment rate of *P. limnetica* (Fig. 9), resulting in only 10% of contaminant cells in culture at 36 ppt, compared to 60–70% at 11 and 18 ppt on day 8. After 16 days, *P. limnetica* completely dominated cultures at 11 and 18 ppt (90–95%), and reached ∼70% dominance at 28 ppt, whereas in cultures at 36 ppt, *P. atomus* was still dominant with 55% (Fig. 9). Specific growth rates [μ] for *P. limnetica* development from day 8 to 10 were ∼0.25 in cultures at 11 and 18 ppt and ∼0.6 in cultures at 28 and 36 ppt. Overall specific growth rates [µ] from days 8 to 16 were ∼0.13 in cultures at 11 and 18 ppt and ∼0.25 in cultures at 28 and 36 ppt. This shows that *P. limnetica* at 11 and 18 ppt were in late logarithmic growth around day 8 whereas at 28 and 36 ppt logarithmic growth was just commencing.

## Discussion

### Effect of salinity on growth and nutrient dynamics of *Picochlorum atomus*

Irrespective of salinity, *Picochlorum atomus* exhibited growth patterns typical of aerated batch cultures [11]. The data established that *P. atomus* is a euryhaline microalga tolerating freshwater to marine salinities without adverse effects on growth and biomass productivities.

Initial specific growth rates [µ] were slightly lower than in previous reports, however maximum biomass [mg DW L^−1^], maximum cell numbers [cells mL^−1^] and initial volumetric productivities [mg DW L^−1^ day^−1^] were comparable to previous reports using similar cultivation procedures for *Picochlorum* spp./*Nannochloris* spp. (Table 5). Comparisons are however, difficult, as a summary of published biomass at harvest and biomass productivities for *Nannochloris* and *Picochlorum* spp. shows great variability (Table 5). This variation is to be expected [41] and is likely due to a combination of effects, such as species-specific responses and cultivation/environmental parameters, i.e. variable inoculation densities, differing light regimes, cultivation (batch vs. semi-continuous) and productivity calculations (direct vs. indirect) [32], [42]–[44].

**Table 5:** Comparison of growth data and reported ranges in this study with growth data obtained for *Picochlorum* spp./*Nannochloris* spp. under similar^*^ and deviating cultivation conditions.

Species	Specific growth rate [µ]	Cell numbers [cells mL^−1^]	Maximum biomass [mg DW L^−1^]	Volumetric productivities [mg DW L^−1^ day^−1^]	References
*Picochlorum atomus* ^*^	0.21–0.28	∼2.2×10^7^	∼560	∼26–43	this study
*Nannochloris atomus* ^*^	∼0.32–0.38	∼3×10^8^	–	–	[44], [57], [68]
*Nannochloris* spp.*/* *Picochlorum* spp.^*^	0.35–0.44	–	∼330–410	∼40	[6], [8], [58]
*Nannochloris maculata* ^*^	∼0.36	∼1×10^8^	–	–	[47]
*Nannochloris bacillaris* ^*^	∼0.41	∼1×10^7^	–	–	[48]
*Nannochloris* spp.*/* *Picochlorum* spp.	0.17–2.5	3×10^6^–3×10^8^	46–1800	7–320	[42], [43], [47], [58], [62], [69]

The decrease in growth rate during phases II and III (Fig. 1, Table 1) is characteristic of batch cultures [11] and is generally the consequence of individual or combined effects of culture self-shading, nutrient limitation [45] and microalgal/bacterial exudate accumulation [24], [46]. Initially, these factors are unlikely to have a considerable effect on culture development, particularly considering the low inoculation densities, adequate nutrient provision and low bacteria cultures used in this study. However, over culture time, the accumulation of algal exudates followed by increased self-shading and bacterial growth-inhibiting exudates (negative allelopathic interactions) are likely to cause the observed decreasing growth rates. Nutrient limitation is unlikely to have affected growth as cultures were maintained nutrient-replete with high nitrite levels (Fig. 3), indicating cellular nitrogen stores were filled throughout most of the cultivation period [33]. Additionally, culture re-fertilisation on day 5 had no impact on culture growth; also implying cultures were not nutrient limited.

The observed growth patterns for *P. atomus* have direct implications for industrial cultivation, as optimal productivities are achieved in relatively dilute cultures for a brief period. Harvest effort and costs inversely correlate with culture cell densities. Consequently, future studies should investigate whether higher inoculation densities and/or semi-continuous culturing would improve biomass yield and overall productivity. In addition, the accumulation of microalgal/bacterial exudates and their effects on culture development require further investigation, as these may affect water treatment and recycling capacity on industrial-scales.

Nitrogen and phosphorus are essential macronutrients, where the first limiting nutrient reduces microalgal growth rates [45]. Therefore, maximum biomass production requires adequate nutrient availability for each particular species in culture. However, excessive nutrient concentrations in harvest water pose environmental problems and unnecessary costs, unless harvest effluents can be efficiently recycled without compromising culture growth.

Initial nitrate uptake by *P. atomus* was similar at all salinities (except 11 ppt) and comparable to *Nannochloris maculata* [47]. With the exception of cultures at 11 ppt, patterns of nitrite secretion until day 10 can be grouped into high (28 and 36 ppt), intermediate (18 and 8 ppt) and low (2 ppt) salinity patterns, where medium nitrite was highest in low salinity cultures. Medium nitrate depletion resulted in expected nitrite resorption as intracellular nitrogen stores became depleted [33]. Nitrogen fluxes can provide insight into possible osmoregulatory mechanisms, often reflected in changes of biochemical profiles. The production of osmoregulatory solutes, such as proline in response to hyperosmotic stress has been reported for *Nannochloris* sp. [48], which would require higher nitrogen provisions. However, despite the variable nitrite secretion, total nitrogen uptake patterns (except for 11 ppt) were not significantly different. This may indicate that higher nitrite secretion in the lower salinity cultures was potentially due to a slight swelling of cells, increasing cell surface area [49], thereby increasing nitrate uptake. In contrast to nitrate [50], nitrite cannot be stored and is cytotoxic in higher concentrations [11]. Reduction of nitrite to ammonium is limited by nitrite reductase activity (a reaction directly linked to photosynthesis and under circadian control [51]). Thus, when nitrate reduction exceeds the reducing capacity of nitrite reductase, nitrite is secreted.

The significantly higher nitrogen requirements at 11 ppt are difficult to explain. Typically, higher nitrogen is required mainly for growth [11], which is not the case here (Fig. 1) or hypersaline osmoregulation [4], but no significant differences in protein contents were detected. Although this does not exclude the production of osmolytes such as glycine betaine or proline [49], osmoregulatory responses would be expected to be higher at lower salinities, which should result in greater nitrogen requirements at lower salinities. As this was not observed, we hypothesise that 11 ppt may induce a transitional response where known hypo- or hyper-osmoregulatory responses are not induced.

At 11 ppt the biomass contained twice the amount of C18∶1(9) and 2–3% more C18∶2 than at other salinities. Fatty acid changes in diacylglycerol (increases in phosphatidyl inositols and hydrolysis of phosphatidyl choline) and an increase in the fatty acid combinations of C16∶0/C18∶1 and C16∶0/C18∶2 were observed in *Dunaliella salina* as an immediate but transient response to hypo-saline osmotic shock (reducing salinity from 99 to 49 ppt) [52]. This indicates that salinity can affect membrane composition. Hence, 11 ppt could induce changes in membrane lipids, perhaps increasing vacuolar storage capacity for nitrogen, which would explain the rapid uptake and the reduced nitrite secretion at 11 ppt.

Nitrate uptake of *P. atomus* was comparable or higher than reported for other species examined for wastewater treatment, including *Chlorella vulgaris* [53] and *Neochloris oleabundans* [54], suggesting that *P. atomus* could also be used in such applications. Nitrogen uptake potential also has important implications for industrial NO flue gas remediation. *Dunaliella tertiolecta* can remediate 21 mg day^−1^ of nitric oxide (NO) and showed a preference for NO uptake over NO_3_^−^ [55]. Future research should examine *P. atomus*’s nitrogen preferences and NO remediation potential from flue gas emitted by Australian coal-fired power stations.

As for nitrate uptake, initial phosphate uptake across all salinities was comparable to *Nannochloris maculata* [47] and uptake rates were comparable to *Chlorella stigmatophora*, showing potential for urban waste-water remediation [56]. Remediation studies using *Neochloris oleabundans* have shown phosphate uptake to correlate with increasing medium phosphate availability [54]. Consequently, further studies should investigate *P.atomus* phosphate uptake when exposed to higher concentrations.

The N:P ratio of *P. atomus* was similar to *Nannochloris atomus* [57]. The N:P ratio decreased over culture time as nutrient availability per cell decreased and cell numbers increased. Downstream effects of the decreased N availability resulted in reduced total protein contents (Fig. 8).

### Effect of salinity and culture nutrient status on the biochemical profile of *Picochlorum atomus*

Culture salinity affected total lipid (at 2 ppt) and protein (at 8 ppt) contents of *Picochlorum atomus* under nutrient-replete conditions. However, nutrient availability was the main driver for significant differences in total lipid, carbohydrate, and protein contents, as well as fatty acid composition. Biochemical profile comparisons between studies are difficult, as species-specificity and environmental factors (nutrient availability, light intensity, photoperiod and cultivation stage) individually and combined affect the proximate chemical composition of microalgae [58]–[60]. Despite being a marine species, the highest total lipid content was observed when culturing *Picochlorum atomus* at 2 ppt, irrespective of nutrient status. Under nutrient-replete conditions, total lipid content of *P. atomus* was low, whereas nitrogen limitation increased total lipids to ∼20%, corresponding to amounts reported for *Nannochloris atomus* and *Picochlorum* sp. [42], [58] and defining it as an oleaginous microorganism with the potential for oil-based biofuel production [61]. In contrast, a higher total lipid content was reported for *Nannochloris* sp. (∼ 56%) when CO_2_ was added [43]. Opportunistic biochemical profiling of very old cultures showed that *P. atomus* can also reach a total lipid content of ∼60%. Consequently, studies should investigate high lipid yields in the context of remaining feasible and economically viable on a large-scale.

Total lipid content is not a good indicator for oil-based products, as this fraction contains all other lipid-soluble materials such as pigments. For oil-based products (e.g. biodiesel and bioplastics), the fatty acid content is more important [37], [41]. Nutrient-depletion increased fatty acid content by ∼10%, suggesting that fertilisation adjustments can improve biomass suitability for such products. Fatty acid proportions of total lipids were comparable to (nutrient-replete) or higher (nutrient-deplete) than those reported for the same genus [42]. Fatty acid profiles were comparable to those described by Volkman et al. [62] but different to others for this genus [42], [44], [58] (which also differ between each other for many fatty acids). These outcomes highlight the importance to consider culture conditions (e.g. industry location) and species-specificity when considering industrial cultivation. Total fatty acid productivities by *P. atomus* were comparable to other species (e.g. *Nannochloropsis* sp.) (see Lim et al. [41] for summary details).

Nutrient limitation considerably increased amounts of saturated (C16∶0) and mono-unsaturated fatty acids (C18∶1) but lowered amounts of polyunsaturated fatty acids (C18∶3) consistent with responses reported for a wide variety of microalgal species [57]. For nutritional/dietary purposes an ω6∶ω3 ratio of approximately 1∶1 has been shown to be beneficial for cardio-vascular health [63], suggesting, that under the cultivation conditions reported here, *P. atomus* should be harvested when nutrient-deplete. In contrast, the suggested optimal fatty acid ratio for biofuel of 5∶4∶1 of C16∶1, C18∶1 and C14∶0, respectively [64] was observed only under nutrient-replete conditions and low concentrations were observed. Identifying species with naturally occurring favourable fatty acid ratios for specific end-products could prove impossible under industrial conditions, therefore blending of fatty acids or oils from different microalgal species [65] and/or fertilisation regimes must be considered to achieve the specifications of a particular end-product. For example, for biofuel production, cultures of *P. atomus* will require nutrient starvation to increase lipid productivity and decrease the PUFA content.

Nutrient status also affected total carbohydrate and protein contents which increased and decreased, respectively, following nutrient limitation. Both carbohydrate and protein contents were similar under nutrient-replete conditions and slightly higher than reported for *Nannochloris atomus* under nutrient limitation [58]. Similar patterns of protein decrease and concurrent carbohydrate increase as a result of nutrient depletion have been observed in a number of microalgal species e.g. *Chlorella vulgaris* and *Scendesmus obliquus* [59], as N-limitation prevents the synthesis of proteins, channelling the photosynthetically acquired carbon into storage. Nutrient-replete *Picochlorum atomus* has been shown to be a promising replacement for *Nannochloropsis oculata* in aquaculture for grouper larval rearing [6], which is rapidly expanding, and already one of the most valuable aquaculture species in Southeast Asia [66].

#### Contaminant inhibition

In large-scale cultures, contamination by rogue organisms is a serious problem often resulting in significant economic losses [67]. In tropical Australia, the freshwater cyanobacterium *Pseudanabaena limnetica* rapidly out-competes and dominates other microalgal species in culture. The observed broad salinity tolerance of *P. atomus*, with minimal effects on productivity or biochemical profiles, allows the use of salinity manipulations to inhibit or reduce culture contamination by rogue organisms. Although increased culture salinity does not completely prevent the development of *P. limnetica*, it does delay its establishment and subsequent logarithmic growth at 28 and 36 ppt up to day 8. It is noteworthy however, that while establishment of *P. limnetica* at high salinities is considerably slower, once established, growth rates are high and culture take-over will occur. The extended time for establishment and logarithmic growth of *P. limnetica* provides an extended opportunity to harvest the biomass with low levels of contamination, which is an important aspect for end product quality control.

In conclusion, *Picochlorum atomus* has considerable advantages for large-scale cultivation as it can be cultivated at locations differing in water salinity ranging from 2 – 36 ppt, without adverse effects on biochemical profiles. High carbohydrate and protein content suggests use in aquaculture [8] or as agricultural feed (e.g. for poultry) [5], when harvested under nutrient-replete conditions. In contrast, under nutrient-deplete conditions, fatty acid yields and the decrease in PUFA content is suitable for lipid-based biofuel production. Similarly, the improved ω6∶ω3 ratio under these conditions, would allow cultivation of *P. atomus* as a health food supplement to improve cardio-vascular health. In addition, salinity increase appears to be an effective tool for contamination delay, yielding biomass with guaranteed quality, which allows harvest and minimises economic losses due to culture re-establishment and end-product loss.
